# Doctor Tanner’s Fast

**Published:** 1880-08-20

**Authors:** 


### *DOCTOR TANNER'S FAST.*

So far as we can ascertain, the recent extraordinary fast of Dr. Tanner in New York, was bona fide and conducted fairly. If so, it furnishes the scientific world with the fact that it is possible, under favorable circumstances, for a man of strong will and good physical constitution, to abstain from food for a period of forty days. We say under favorable circumstances, because the instances are numerous in which parties deprived of food have survived for a period of only a few days.

Many years ago the steamship Medusa was disabled with 150 on board who remained upon the vessel thirteen days before discovered, without food or drink. Only 15 out of the 150 were alive at the end of 13 days. Usually on the fifth or sixth day delirium sets in, the sufferer rapidly sinks and dies.

In the Tanner case water was allowed, and the fact is shown that water and atmospheric air are more important factors in the sustenance of the human frame than heretofore supposed.

It would be a great comfort to know absolutely whether or not the experiment was fairly conducted. Upon this point we were very skeptical until we received the subjoined letter from Dr. Low, an intelligent medical gentleman with whom we are intimately acquainted, and who was one of the watchers in the case, since which we have confidence in the fairness of the experiment.

The letter was written on the 30th day of the fast. Subsequent reports showed but little change in the symptoms, save an increase in the frequency of the attacks of vomiting and the increased debility.

The first food taken was a glass of milk followed by a portion of water mellon and the chewing of large quantities of beefstake, and swallowing the substance. Wine was also taken and, after a day or two, stewed potatoes and other food in great abundance, from which he rapidly recuperated and gained flesh wonderfully fast. W.

*New York City, July* 27, 1880.

DEAR DOCTOR: I passed to-day four hours with Dr. Tanner. The Doctor is an Englishman by birth, forty-nine years old, about five feet six inches in height, symmetrically formed, of a bilious, nervous temperament, and while, I think, under ordinary circumstances, would be genial and good disposed, is, from his long fasting, rather irritable.

He is in Clarendon Hall, situated on Thirteenth Street. It is quite a spacious Hall with a rostrum of six feet wide across its entire width both in front and rear, and on the latter of which lies the Doctor on his cot, surrounded by his watch, while below, in the large reception hall sit the visitors, and on the front rostrum is a piano, and he is favored with music by both ladies and gentlemen visitors, and not unfrequently by full bands of music, which the Doctor seems to enjoy very much and evinces his appreciation by the clapping of his hands.

The Doctor, for a number of days at the commencement of his fasting, drank no water, from which cause he complained of great nausea which afterward was relieved by small draughts of water, while large drinks of water would bring on the nausea.

I took the Doctor a paper. He does not read anything himself, or at least very little, but has the papers read to him daily. He takes morning and evening rides in the Central Park of about ten miles, always attended with three of the watch. His watch is composed of doctors of the various schools, allopathic, eclectic and homeopathic. The Doctor, about two o'clock, walked down stairs, with a firm step to the audience room, was weighed—weight 130 pounds—losing on an average about one pound per day, original weight 157½ pounds, this being his thirtieth day. He occupied his lower cot about half an hour and returned up the steps to his cot on the rostrum—his upper cot.

The specific gravity of his úrine is to-day 1010; it has been as high as 1030, and as low as 1004, varying, of course, according to the quantity of water drank. Tested to-day with the nitrate of silver, the urine throwed down the nitrate of lime, showing clearly too much phosphate of lime in the urine. He had been drinking some, as was supposed, very pure water that had been sent him, but when analyzed proved to contain a superabundance of lime. A great deal of water is sent to the Doctor, supposed to be by the donors very pure, but it is tested thoroughly.

The Doctor has not had an action on his bowels for the thirty days, and says he has gone before as long as forty-seven days without one. The large quantity of urea in the urine goes to prove that he has taken no food.

The regurgitation of the bile causes sickness of the stomach, which he says he will continue to have until the expiration of the forty days. He is troubled with flatulency; and the gas passes up and down, both night and day, which affords his stomach great relief.

It is now claimed that he has thirty pounds more of flesh that he can lose and still live. He drank in the four hours yesterday ℥j and ʒj of water, bathed his head once in rain water, and slept one hour during the time.

His strength was tested on yesterday by the dynamometer; he pressed down 85 with his left hand and only 80 with his right hand. Upon a second trial with his right hand he fell back a point or two.

His respiration at two o'clock was 14. His circulation tested by the sphygmograph indicated 84 per minute, and more normal than it had been for several days. Temperature 98.

You would be surprised to see how well he keeps up under all the circumstances. His complexion is fine, and he seems to have that degree of will and nerve power that will bear him up or sustain him until the expiration of the forty days.

